# Faster cognitive decline in the years prior to MR imaging is associated with smaller hippocampal volumes in cognitively healthy older persons

**DOI:** 10.3389/fnagi.2013.00021

**Published:** 2013-06-05

**Authors:** Debra A. Fleischman, Lei Yu, Konstantinos Arfanakis, S. Duke Han, Lisa L. Barnes, Zoe Arvanitakis, Patricia A. Boyle, David A. Bennett

**Affiliations:** ^1^Rush Alzheimer's Disease Center, Rush University Medical CenterChicago, IL, USA; ^2^Department of Neurological Sciences, Rush University Medical CenterChicago, IL, USA; ^3^Department of Behavioral Sciences, Rush University Medical CenterChicago, IL, USA; ^4^Department of Diagnostic Radiology and Nuclear Medicine, Rush University Medical CenterChicago, IL, USA; ^5^Department of Biomedical Engineering, Illinois Institute of TechnologyChicago, IL, USA; ^6^VA Long Beach Healthcare SystemLong Beach, CA, USA

**Keywords:** neuroimaging, macrostructure, hippocampal volume, aging, dementia, Alzheimer's disease, mild cognitive impairment, Rush Memory and Aging Project

## Abstract

Early identification of persons at risk for cognitive decline in aging is critical to optimizing treatment to delay or avoid a clinical diagnosis of mild cognitive impairment (MCI) or dementia due to Alzheimer's disease (AD). To accomplish early identification, it is essential that trajectories of cognitive change be characterized and associations with established biomarkers of MCI and AD be examined during the phase in which older persons are considered cognitively healthy. Here we examined the association of rate of cognitive decline in the years leading up to structural magnetic resonance imaging with an established biomarker, hippocampal volume. The sample comprised 211 participants of the Rush Memory and Aging Project who had an average of 5.5 years of cognitive data prior to structural scanning. Results showed that there was significant variability in the trajectories of cognitive change prior to imaging and that faster cognitive decline was associated with smaller hippocampal volumes. Domain-specific analyses suggested that this association was primarily driven by decline in working memory. The results emphasize the importance of closely examining cognitive change and its association with brain structure during the years in which older persons are considered cognitively healthy.

## Introduction

Early identification of persons at risk for cognitive decline in aging is critical to optimizing treatment to delay or avoid a clinical diagnosis of dementia due to Alzheimer's disease (AD; Sloane et al., 2002; Cummings et al., 2007). Accordingly, the diagnostic continuum of AD has recently been re-conceptualized to emphasize clinical AD diagnosis as the final stage of the disease, and attention has shifted to characterizing cognitive and biologic markers in a preclinical phase that can reliably identify those persons at increased risk of ultimately developing clinical AD (Jack et al., 2011; Sperling et al., 2011).

This preclinical phase, during which subtle cognitive changes are occurring in some cognitively healthy older persons, can be quite long, potentially lasting many years (Amieva et al., 2008; Johnson et al., 2009; Wilson et al., 2011, 2012). Cognitively healthy persons who are experiencing subtle cognitive decline within the normal range may be undergoing a clinically-silent pathological cascade of brain changes during this phase (Mormino et al., 2008; Jack et al., 2010a,b; Bennett et al., 2012b). One of those brain changes, hippocampal atrophy, is thought to occur late in this cascade (Sperling et al., 2011) and, when it is associated with overt cognitive impairment, may mark the transition out of the preclinical phase. Indeed, the association between hippocampal atrophy and mild cognitive impairment (MCI) and AD is a well-established finding in cross-sectional and prospective longitudinal studies (e.g., Mungas et al., 2002; Chetelat and Baron, 2003; Jack et al., 2010b; Risacher et al., 2010; Sperling et al., 2011; Van Rossum et al., 2012).

The association between hippocampal atrophy and cognitive function in cognitively healthy persons in both cross-sectional and prospective longitudinal studies, however, is not as clear. Medial-temporal, including hippocampal, volume loss has been reported and associated with cognitive function in a number of studies of older persons considered cognitively normal (e.g., Mu et al., 1999; Tisserand et al., 2000; Raz et al., 2005; Mungas et al., 2005; Du et al., 2006; Tupler et al., 2007; Head et al., 2008; Dickerson et al., 2009; Raji et al., 2009; Fjell et al., 2010b; Walhovd et al., 2011; Rosano et al., 2012), but just as many other studies find that this association is weak or not present (e.g., Raz et al., 1997; Good et al., 2001; Resnick et al., 2003; Salat et al., 2004; Van Petten, 2004; Allen et al., 2005; Fjell et al., 2009, 2010a; Lo et al., 2011; Lemaitre et al., 2012).

There are at least three important reasons for the mixed findings. First, studies do not always distinguish between subjects who are and are not declining in cognitive function within the normal range. As a result, samples across studies may differ in the number of subjects included that are considered cognitively healthy but may be declining. Second, in longitudinal studies, the number of data collection time points is often limited. Given the length of the preclinical phase, multiple years of testing may be needed to identify those persons that are declining cognitively. Third, in a cognitively healthy group, there can be ceiling effects in cognitive function depending on the scale chosen to measure it. For example, ceiling effects have been demonstrated for AD patients on the frequently used Alzheimer's Disease Assessment Scale-cognitive subscale (ADAS-cog), an 11-item summary measure (Hobart et al., 2013), and it is quite likely that the scale is not sensitive to subtle compromise or change in cognitive function within the normal range (Hobart et al., 2013). Further, using a single measure of global cognitive function limits what can be learned about specific aspects of cognitive function and cognitive decline that may be associated with brain integrity. Thus, there is a need for a longitudinal study that examines the association between hippocampal atrophy, a well-established biomarker for MCI and AD, and change in cognition during the phase when older persons are considered cognitively healthy, across many cycles during this protracted phase, using measures of global and specific domains of cognition that are known to be sensitive to change within the normal range (Wilson et al., 2011, 2012).

In this study, we examined cognitive change and its association with hippocampal atrophy during the cognitively healthy years leading up to structural imaging. The Rush Memory and Aging Project, a longitudinal cohort study of aging and dementia began in 1997 and introduced neuroimaging in 2009. Therefore, we were able to measure the rate of global cognitive change, as well as the rate of change in five specific cognitive systems, in multiple years leading up to structural brain imaging in clinically well-characterized persons who did not have MCI or dementia at time of scan. We tested the hypothesis that older persons who experienced faster decline in cognition, but who were still considered cognitively healthy at the time of scan, would have smaller hippocampal volumes.

## Materials and methods

### Subjects

The subjects are participants in an on-going longitudinal cohort study of aging and dementia. The Rush Memory and Aging Project, which began in 1997, has a rolling admission and requires annual clinical evaluation and brain donation at death (Bennett et al., 2012a). Neuroimaging was introduced into the study in 2009. The study is approved by the institutional review board of Rush University Medical Center.

At the time of analyses, 1528 participants had enrolled and completed their baseline evaluation: 564 died, 107 refused further participation in either the parent study or neuroimaging substudy before scan data could be collected, and 342 were not eligible for the scan due to various reasons including MRI contraindications. Of the remaining 515, 440 were scanned and 75 were being scheduled for scanning. Of the 440 that were scanned, 414 had data on hippocampal volume, of which 8 were demented at scan and 138 had only one cognitive data point prior to scan. Of the remaining 268, 57 had MCI and 211 were cognitively normal.

### Clinical evaluation

All participants underwent an annual uniform and structured clinical evaluation that included a medical history, complete neurological examination, and cognitive performance testing. Based on these data and in-person evaluation of the subjects, an experienced clinician diagnosed dementia and AD using the criteria of the joint working group of the National Institute of Neurologic and Communicative Disorders and Stroke and the AD and Related Disorders Association (McKhann et al., 1984). The criteria require a history of cognitive decline and impairment in at least two cognitive domains, one of which must be memory for a diagnosis of AD. As previously described (Bennett et al., 2012a), impairment in five cognitive domains (orientation, attention, memory, language, and visuospatial ability) was determined in a two-step process. First, an algorithm rated impairment in each domain based on educationally-adjusted cutoff scores on 11 individual tests. Second, based on all test data and information on education, sensorimotor problems, and effort, a neuropsychologist agreed, or disagreed with each rating and supplied a new rating in the event of disagreement. Persons who had cognitive impairment but did not meet criteria of dementia were classified as having MCI. All clinical classification was done blinded to previously collected data.

### Cognitive evaluation

A battery of 21 cognitive performance tests was administered in an approximately hour-long session during baseline and annual follow-up sessions. The Mini-Mental State Examination (MMSE) was used as an overall measure of cognitive ability and one test (Complex Ideational Material) was only used for diagnostic classification (Bennett et al., 2012a). Episodic memory measures included Word List Memory, Word List Recall and Word List Recognition from the procedures established by the CERAD, immediate and delayed recall of Logical Memory Story A and the East Boston Story. Semantic memory measures included Verbal Fluency, Boston Naming, and a subset of items from the National Adult Reading Test. Working memory measures included the Digit Span subtests (forward and backward) of the Wechsler Memory Scale-Revised and Digit Ordering. Measures of perceptual speed included the oral version of the Symbol Digit Modalities Test, Stroop Test, and Number Comparison. Measures of visuospatial ability included Judgment of Line Orientation and Standard Progressive Matrices. Raw scores on each test were converted to standard z scores using the mean and standard deviation from the baseline evaluation. A person's standard scores across 19 tests were averaged to yield a single overall cognitive composite score. A composite score for five cognitive domains (episodic memory, semantic memory, working memory, perceptual speed, visuospatial ability) was created by averaging the *z*-scores of all measures within a domain (Bennett et al., 2012a).

### MRI acquisition and post-processing

MRI scans were performed on a 1.5 Tesla General Electric MRI scanner (GE, Waukesha, WI). High-resolution T_1_-weighted anatomical data was obtained for all subjects using a 3D magnetization-prepared rapid acquisition gradient-echo (MPRAGE) sequence with the following parameters: TE = 2.8 ms, TR = 6.3 ms, preparation time = 1000 ms, flip angle 8°, field-of-view 24 cm × 24 cm, 160 sagittal slices, 1 mm slice thickness, no gap, 224 × 192 image matrix reconstructed to 256 × 256, scan time = 10 min and 56 s. Two copies of the T_1_-weighted data were acquired on each subject. The two T_1_-weighted datasets from each subject were co-registered using rigid-body registration and averaged. The average T_1_-weighted dataset of each subject was then segmented using FreeSurfer Version 5 (http://surfer.nmr.mgh.harvard.edu; Fischl et al., 2002; Xiao and Fischl, 2007). The results were reviewed and any errors were manually corrected. The volume of the segmented gray matter regions, including the hippocampus, and the total intracranial volume (ICV) were estimated for each subject. The volumes of homologous regions in contralateral hemispheres were averaged. Finally, hippocampal and all other volumes were normalized by dividing by the total ICV.

Daily quality assurance tests were conducted on the scanner according to the American College of Radiology (ACR) protocol. The data from these tests were used to evaluate the performance of the MRI scanner hardware on days data on human subjects was collected. An algorithm developed in-house was used to automatically produce the results of the quality assurance tests. Furthermore, the whole brain signal to noise ratio (SNR), and the contrast to noise ratio (CNR) between white and gray matter were estimated for the average T_1_-weighted dataset of each subject. The mean and standard deviation of the SNR and CNR were calculated over all subjects. Individual datasets with SNR or CNR lower than two standard deviations from the mean SNR and CNR over all subjects were inspected for potential problems in the raw data or with automated brain segmentation. Finally, the data were inspected for outliers in ICV-normalized volumes.

### Statistical approach

To investigate the temporal association between the pre-scan rate of change in cognition with total hippocampal volume, we first estimated the slope of cognitive decline for each individual by fitting a linear mixed model to all available longitudinal cognitive testing data up until the time of neuroimaging, adjusted for age, sex, and years of education. These person-specific slopes were then used in ordinary linear regression as the predictor for hippocampal volume. All subsequent regression models were also adjusted for age at scan, sex, and education.

We first examined the association of pre-scan rate of change in global cognition with hippocampal volume. Next, because it is possible that the association may differ across cognitive domains, in subsequent analyses, we examined the association of the rates of change in five different cognitive domains of episodic memory, semantic memory, working memory, perceptual speed, and visuospatial ability with hippocampal volume. Finally, because the presence of vascular disease burden or vascular disease risk might influence the association of pre-scan change in cognition with the hippocampal volume, in secondary analyses we augmented our core model by adding covariates for vascular disease burden (a composite score of vascular diseases including claudication, stroke, heart conditions, and congestive heart failure) as well as vascular disease risk (a composite score of vascular disease risk factors including hypertension, smoking and diabetes). We imposed a nominal threshold of *p* < 0.05 for statistical significance and all analyses were implemented using SAS® software, version 9.3 (SAS Institute Inc., 2012).

## Results

Two hundred and eleven subjects participating in the Rush Memory and Aging Project were included in the analysis. Descriptive characteristics of the group are provided in Table 1. The mean age at scan was 82.7 years (*SD* = 6.7, range = 65.2–100.3). The average length of follow-up prior to imaging was 5.5 years (*SD* = 2.7, range = 0.6–13.0). Comparing this sample with the subjects that were scanned but were excluded from the analysis due to dementia or lack of follow-up data, we found no significant differences between the two samples in age, sex, or education. Subjects that were excluded from the analysis had, on average, lower global cognitive function (*p*-value < 0.001) and fewer vascular diseases (*p*-value < 0.001). The difference in vascular disease risk was marginal (*p*-value = 0.086).

**Table 1:** **Descriptive characteristics of the participants in the study (*N* = 211)**.

	**Mean (*SD*)/*N* (%)**
Age at scan (years)	82.7 (6.7)
Education (years)	15.3 (3.1)
Female	154 (73.0%)
MMSE at scan	28.7 (1.3)
Global cognitive score at scan	0.38 (0.44)
Total hippocampal volume (× 10^−3^)	4.1 (0.7)
Pre-scan follow-up years	5.5 (2.7)
At least one vascular disease	82 (38.9%)
At least one vascular risk factor	170 (80.6%)

Quality assurance testing demonstrated that scanner performance was satisfactory and consistent. The SNR and CNR of all datasets included in this study exceeded the minimum acceptable limits. No outliers in ICV-normalized hippocampal volumes were identified.

Average ICV-normalized hippocampal volume was 4.1 × 10^−3^ (*SD* = 0.7 × 10^−3^, range = 2.7 × 10^−3^ to 6.5 × 10^−3^). Simple correlation analyses revealed that hippocampal volume was negatively associated with age at scan (*r* = −0.57, *p* < 0.001) and positively associated with global cognition at scan (*r* = 0.26, *p* < 0.001) and MMSE score (*r* = 0.26, *p* < 0.001). Hippocampal volume was not related to years of education. In this group, females had larger hippocampal volumes than males (Diff = 0.36 × 10^−3^, *t* = 3.87, *df* = 128.3, *p* < 0.001).

**Figure 1:**
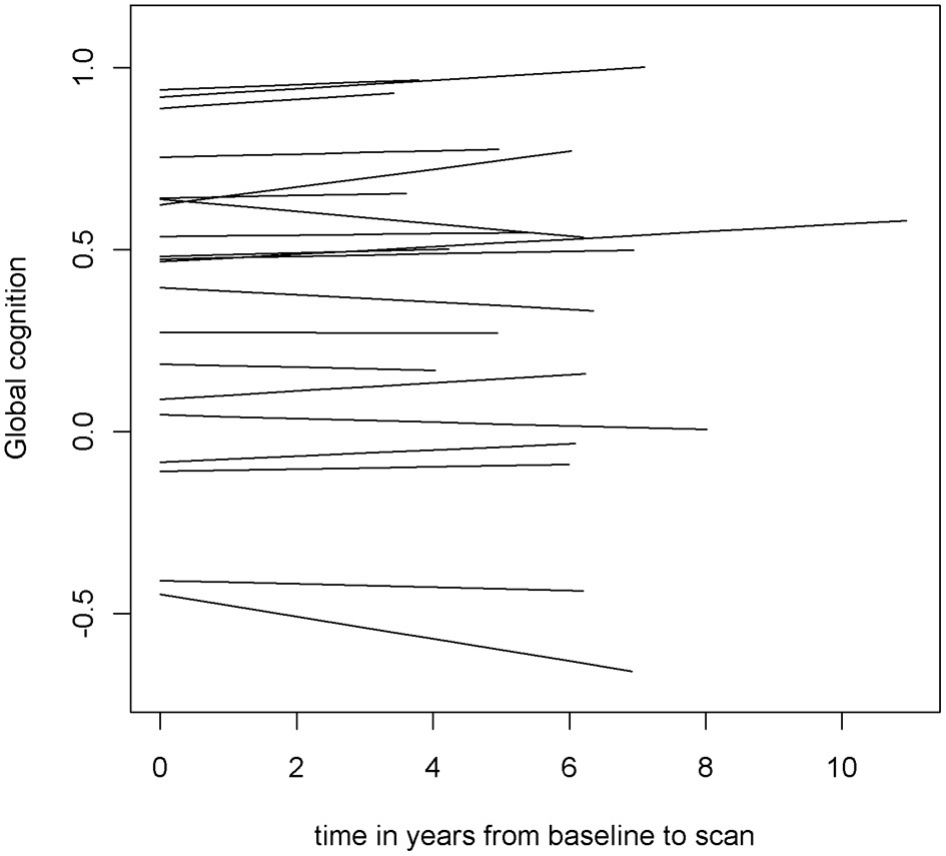
**Predicted linear decline in global cognition for a randomly-selected sample of 20 persons**.

**Figure 2:**
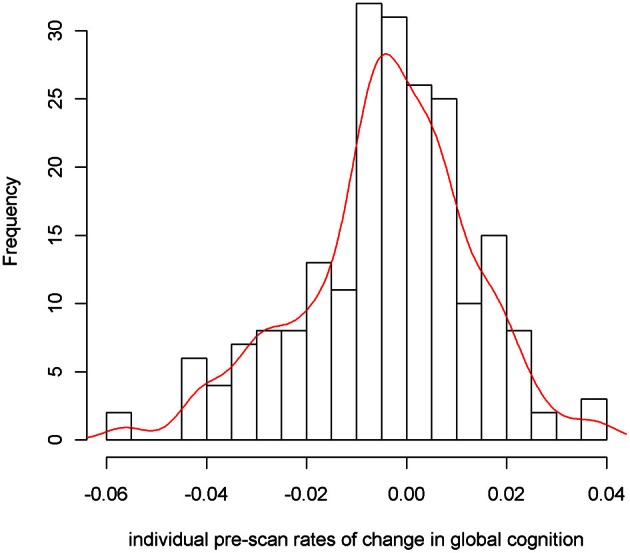
**Distribution of person-specific pre-scan rates of decline**.

To examine the association of pre-scan rate of change in cognitive function with hippocampal volume, we first constructed a linear mixed-effects model with a term for time (since baseline in years) to estimate each person's annual rate of global cognitive change (i.e., slope), adjusted for age, sex, and education. On average there was no overall decline in global cognition (Estimate = −0.004, *SE* = 0.004, *p* = 0.273). However, since some persons declined, others remained the same, and still others improved as a result of practice and learning, the variance estimate for person-specific slopes was highly significant (Estimate = 0.0007, *SE* = 0.0002, *p* < 0.001). Figure 1 illustrates the predicted linear decline in global cognition for a randomly-selected sample of 20 persons. Figure 2 further illustrates the distribution of person-specific pre-scan rates of decline (Mean = −0.005, *SD* = 0.019, range = −0.057 to 0.039). As shown by these figures, even among these cognitively healthy persons, there is a sizeable amount of variability in the rates of decline: some persons declined faster, some slower, while other persons improved slightly.

**Figure 3:**
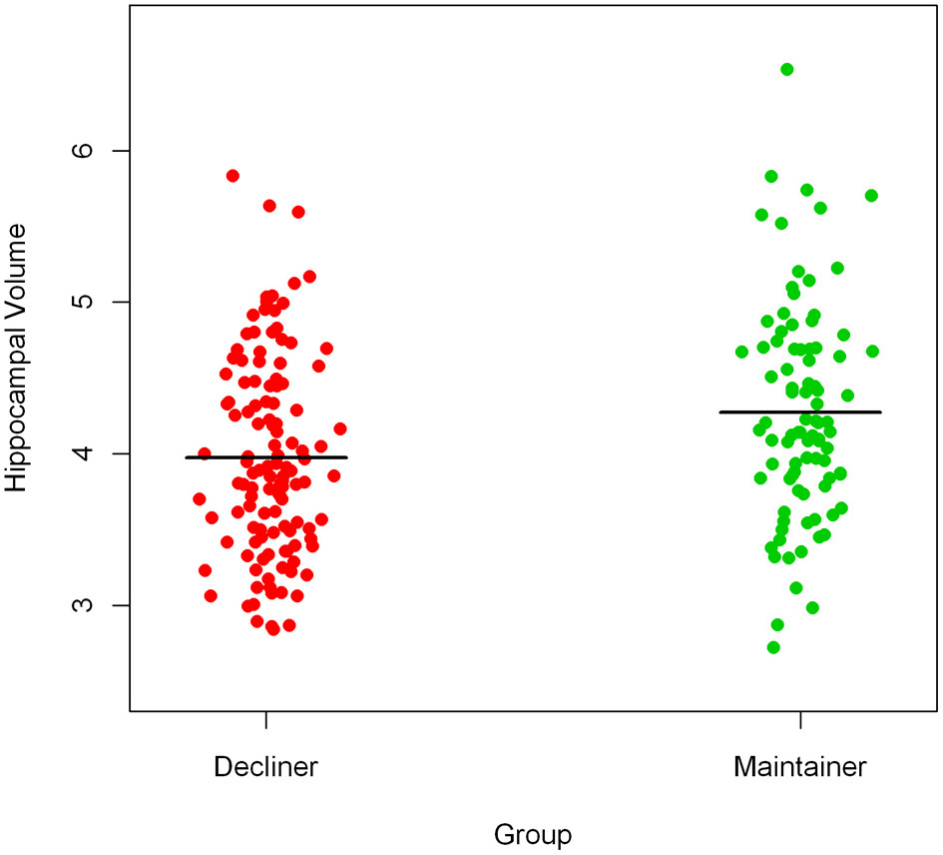
**Mean difference in hippocampal volume between cognitive decliners and cognitive maintainers**.

To illustrate the difference in hippocampal volumes between cognitively healthy persons with declining slopes (cognitive decliners, 57.8% of the sample) and cognitively healthy persons with non-negative slopes (cognitive maintainers, 42.2% of the sample), we present a prism-like plot showing the mean difference in hippocampal volume between the groups. Figure 3 shows that persons who declined in cognitive function prior to time of scan had on average smaller hippocampal volumes.

Next, to formally test the hypothesis that faster rate of decline in global cognitive function prior to scan was associated with smaller hippocampal volume, we constructed a linear regression model with hippocampal volume as the outcome and terms for global cognitive slope, age at scan, sex, and education as the predictors. The result of this analysis showed that a more rapid rate of pre-scan cognitive decline was associated with smaller hippocampal volume (*p* = 0.019; Table 2). To clarify the magnitude of this effect, when the rate of pre-scan decline in global cognition increased by 1 standard deviation, the average reduction in total hippocampal volume was equivalent to an increase of about 2 years of age. The results were unchanged after adjusting for vascular disease burden and vascular disease risk.

**Table 2:** **Pre-scan rate of change in cognition and hippocampal volume**.

**Cognitive measures**	Estimates^*^ (*SE*), *p*
Global cognition	0.055 (0.023), 0.019
Episodic memory	0.033 (0.017), 0.059
Semantic memory	0.042 (0.045), 0.347
Working memory	0.065 (0.027), 0.017
Perceptual speed	0.017 (0.010), 0.112
Visuospatial ability	−0.106 (0.129), 0.414

* Estimates refer to the increase in hippocampal volume (× 10^−3^) with every 0.01 unit increase in rate of change in cognition. All the models were adjusted for age at scan, sex, and years of education.

Finally, we examined the association of rates of change in domain-specific summary measures of cognition with hippocampal volume. Analyses of five different cognitive domains indicated that smaller hippocampal volume was associated with faster pre-scan decline in working memory (*p* = 0.017). There was also a strong trend for smaller hippocampal volume to be associated with pre-scan decline in episodic memory (*p* = 0.059). Hippocampal volume was not associated with pre-scan decline in semantic memory, perceptual speed or visuospatial ability.

## Discussion

The results of this study demonstrate that when cognition is fully characterized over a sufficient period of time during the phase when older persons are considered cognitively healthy, substantial individual variability in slopes of cognitive change is observed and a faster rate of cognitive decline, particularly in working memory, can be linked to hippocampal atrophy, a well-established biomarker of risk for MCI and/or AD.

Our findings are consistent with a number of studies that report cognitive decline in the healthy years preceding a clinical diagnosis of MCI and/or AD (e.g., Amieva et al., 2008; Johnson et al., 2009; Wilson et al., 2011, 2012; Rosano et al., 2012) and extend these results by underscoring the substantial variability in cognitive function that occurs within the normal range during these years. It is clear that some persons decline, some stay stable and others improve, and this heterogeneity may be one explanation for mixed findings regarding the relationship of cognitive decline to measures of brain integrity in the cognitively healthy years. In this study, those persons who were cognitively healthy at the time of scan, but who declined cognitively in the years preceding the scan, had smaller hippocampal volumes.

We measured cognitive decline globally, but also in five different domains, and found that the association with smaller hippocampal volume was driven most strongly by decline in working memory. This finding is in line with studies that have connected the soundness of working memory in aging to the integrity of the hippocampal region (reviewed in Salthouse, 2011). However, the association between episodic memory and hippocampal atrophy was weaker, a finding that is often noted in studies of cognitively healthy older persons (reviewed in Van Petten, 2004). When we separated our sample into domain-related decliners and maintainers, the percentage of working memory-decliners was quite high (97%) and the association with hippocampal atrophy was strong, whereas the percentage of episodic memory-decliners was quite low (14%) and the association with hippocampal atrophy was marginal. Again, these findings emphasize the importance of addressing sample composition in longitudinal studies of cognition and brain integrity in cognitively healthy older persons. Most importantly, however, they suggest that older persons who are considered cognitively healthy but have evidence of cognitive decline, particularly in working memory, may be amid a pathological cascade and on a protracted trajectory toward neuronal injury, episodic memory impairment and eventually a clinical diagnosis of MCI or AD.

It has been established in many studies that maintaining cognitive function in older age lowers the risk of adverse cognitive and functional outcomes (reviewed in Hertzog et al., 2009), however, the association of cognitive maintenance with brain integrity is not well-studied. Only one study that we know of has examined the relationship of cognitive maintenance to brain integrity in cognitively healthy older persons in the years prior to imaging. Rosano et al. (2012) reported that 59% of persons in their sample maintained global cognitive function, based on the Modified Mini-Mental State Examination (3 MS; Teng and Chui, 1987), over 4 time points in the decade prior to time of scan. Cognitive maintainers had larger medial temporal lobe (hippocampus, parahippocampus, entorhinal cortex) gray matter volumes. The results of the current study support this finding in that global cognitive maintainers (42.2% of our sample) had larger hippocampal volumes compared to cognitive decliners. However, the possibility that cognitive maintenance reflects susceptibility to practice effects needs to be addressed. We examined this possibility in secondary analyses using scores from the cognitive domain that generated the largest percentage of cognitive maintainers, episodic memory (86%). We added additional terms to the linear mixed models representing the number of follow-up years of cognitive testing, as previously reported (Wilson et al., 2006). We found some evidence of a practice effect on episodic memory, however, the percentage of episodic memory maintainers still reached 60% after adjusting for this practice effect. This suggests that these individuals are genuinely maintaining or improving their episodic memory. A number of lifestyle behaviors that can potentially protect cognition have been examined (reviewed in Hertzog et al., 2009). For example, it has been shown that frequent mental stimulation leads to better cognitive function (Wilson et al., 2012), particularly in episodic memory. More studies are needed to further understand the brain basis of this phenomenon.

This study has important strengths. The data were sampled from a large, longitudinal clinical-pathological study in which subjects have participated in up to 14 annual assessments using well-established clinical and cognitive measures. The study also has limitations. The period of time over which cognitive change was measured in this study cannot be considered preclinical. All subjects were cognitively healthy at time of scanning and we await clinical outcomes. Although the Rush Memory and Aging Project was designed to closely represent the general population of persons aged 65 and over, the sample in this study was selected. Multiple years of cognitive data allowed the examination of cognitive change, but the volume data are from one time point. Thus, these data cannot address simultaneous change in pre-scan cognition and hippocampal volume. However, participants of the Rush Memory and Aging Study agree to bi-annual scanning until death so it will be possible to examine the associations between cognitive change, transition to clinical diagnoses, and macrostructural change in the future. Hippocampal volume in the elderly may be influenced by the presence of not only AD pathology, but other pathologies such as hippocampal sclerosis (Dawe et al., 2011), Lewy bodies (Burton et al., 2012), and amyloid angiopathy (Jagust et al., 2008; Erten-Lyons et al., 2013). Although we cannot address the neuropathology of reduced volume in this study, histopathologic information will be available for these subjects in the future. Finally, a stronger magnet would have allowed a closer examination of associations with hippocampal volumes in specific subfields. Using a 4T magnet, the CA1 subfield has been shown to be most strongly affected by age, particularly in the seventh decade (Mueller et al., 2006) and hippocampal deformation was primarily attributable to CA1 volume loss in a post-mortem imaging shape analysis of elderly persons over the age of 65 (Dawe et al., 2011). Post-mortem imaging will also be available for these subjects in the future. These limitations notwithstanding, the results of this study are important for at least two reasons. First, they emphasize the need to deeply characterize cognition, brain structure and their relation during the years in which older persons are considered cognitively healthy. Second, the findings are clinically relevant. Whereas clinicians often use imaging biomarkers such as hippocampal volume to predict subsequent cognitive decline, these findings show that hippocampal volume can inform on the trajectory of cognitive change during the period of time preceding the patient's first presentation to the clinic. This information would help the clinician elucidate the patient's cognitive history, identify risk of developing a clinical diagnosis of MCI or dementia due to AD and optimize treatment.

### Conflict of interest statement

The authors declare that the research was conducted in the absence of any commercial or financial relationships that could be construed as a potential conflict of interest.
